# External Financial Aid to Blood Transfusion Services in Sub-Saharan Africa: A Need for Reflection

**DOI:** 10.1371/journal.pmed.1001309

**Published:** 2012-09-11

**Authors:** Fereydoun Ala, Jean-Pierre Allain, Imelda Bates, Kamel Boukef, Frank Boulton, James Brandful, Elizabeth M. Dax, Magdy El Ekiaby, Albert Farrugia, Jed Gorlin, Oliver Hassall, Helen Lee, André Loua, Kathryn Maitland, Dora Mbanya, Zainab Mukhtar, William Murphy, Ohene Opare-Sem, Shirley Owusu-Ofori, Henk Reesink, David Roberts, Oscar Torres, Grace Totoe, Henrik Ullum, Silvano Wendel

**Affiliations:** 1 London, United Kingdom; 2 University of Cambridge, Cambridge, United Kingdom; 3 Liverpool School of Tropical Medicine, Liverpool, United Kingdom; 4 University of Monastir, Monastir, Tunisia; 5 University of Southampton, London, United Kingdom; 6 Noguchi Memorial Institute for Medical Research, University of Ghana, Accra, Ghana; 7 Fitzroy, Australia; 8 Shabrawishi Hospital, Giza, Egypt; 9 University of Western Australia, Crawley, Australia; 10 Memorial Blood Centers, St Paul, Minnesota, United States of America; 11 University of Oxford, Oxford, United Kingdom; 12 University of Guinée, Conakry, Guinea; 13 Imperial College London, London, United Kingdom; 14 University of Yaoundé, Yaoundé, Cameroon; 15 Transfusion Medicine, Karachi, Pakistan; 16 University College, Dublin, Ireland; 17 University of Science & Technology, Kumasi, Ghana; 18 Komfo Anokye Teaching Hospital, Kumasi, Ghana; 19 Academic Medical Center, Amsterdam, The Netherlands; 20 NHS Blood and Transplant, Watford, United Kingdom; 21 Hospital Materno-Infantil Ramón Sardá, Buenos Aires, Argentina; 22 Community Blood Center, Duluth, Minnesota, United States of America; 23 Rigshospitalet, Copenhagen, Denmark; 24 Sirio Libanes Hospital, Sao Paulo, Brazil

## Abstract

Jean-Pierre Allain and colleagues argue that, while unintended, the foreign aid provided for blood transfusion services in sub-Saharan Africa has resulted in serious negative outcomes, which requires reflection and rethinking.

**Summary Points**

- Development aid to sub-Saharan African blood services has brought benefits but also some unintended negative consequences.
- Policies and practices from funding countries, particularly exclusive use of volunteer non-remunerated donors, centralisation, and systematic preparation of blood components are not necessarily appropriate for sub-Saharan Africa where the vast majority of transfusions are done as emergencies.
- Implementation of these policies and practices adds significantly to the cost of a unit of blood, making the transfusion services unaffordable in resource poor settings and creating long term reliance on external funding.
- We argue that externally funded initiatives for strengthening transfusion services in sub-Saharan Africa should be based on appropriate evidence and adapted to take account of the local context, available resources, and long term sustainability.

Over the past ten years, high-income countries in the North have provided considerable financial aid to establish and support national blood transfusion services in low-income countries in sub-Saharan Africa. This action has largely been driven by concerns relating to the contribution of blood transfusion to the HIV epidemic in the region, leading to the overwhelming objective of “safe blood.” Whilst there have been definite benefits to transfusion services, we believe this aid has resulted in unintended but serious negative outcomes, which we describe here and argue should prompt a re-thinking of how to provide support to blood transfusion services in sub-Saharan Africa.

Support has targeted either single countries such as Malawi, Rwanda, Burkina Faso, and Uganda [1],[2] or multiple countries [3]. Funds, available only for limited periods, have been used to support a combination of infrastructure design and construction; purchase of equipment; screening for transfusion-transmitted infections and quality assurance; and the recruitment of blood donors. As a direct consequence of this funding, some of the underlying principles of transfusion services practised in the high-income donor countries have been applied in sub-Saharan Africa recipient countries (Box 1). These principles may be based on sound practice in wealthy countries but do not necessarily apply to sub-Saharan Africa at this time. We argue that considering the needs of sub-Saharan Africa, external aid was to some extent misdirected in the areas of donor recruitment, overall organisation, and availability of products.

**Box 1. The Principles Underlying External Financial Support for Blood Transfusion Services in Sub-Saharan Africa**

- centralisation of operations in relatively large blood centres, which are stand alone institutions independent physically and functionally from hospitals able to collect, process, and distribute >10,000 blood donations a year. Automation becomes feasible and quality assurance programmes are more practicable.
- The exclusive recruitment of VNRD, which has been practiced for over 30 years in developed countries; this is based on the assumption that other types of donor are less safe owing to higher prevalence of HIV-1 infections.
- The preparation of blood components with the assumption that this is a more effective use of whole blood donations.

## Current Transfusion Practice in Sub-Saharan Africa

It is important to recognise the particular circumstances and specific needs of recipient countries, which differ considerably for historical and economic reasons. Whereas blood product use in wealthy countries is largely pre-planned and predictable, the vast majority of blood product use in sub-Saharan Africa is for emergencies, therefore truly saving lives when delivered quickly [4]. Depending on the country, 50% to 80% of transfusions are related to just a few clinical circumstances: severe haemorrhage in women related to pregnancy and childbirth; trauma usually as a consequence of road traffic crashes; and severe anaemia in young children, often due to malaria. Timely access to blood transfusion has a clear role to play in achieving two of the Millennium Development Goals—reducing death rates by two-thirds in children under-five and by three-quarters in mothers. For severe malarial anaemia the product of choice, which has been shown to save lives and is recommended by the World Health Organization (WHO), is whole blood [5]–[8].

Before national blood transfusion services were established, blood provision in sub-Saharan Africa was totally decentralised, with blood banks operating in individual hospitals and often collecting 1,000 to 10,000 units a year. These blood banks were, and still are, an integral part of the hospitals they serve and are located close to patients, clinicians, and hospital managers [9]. Blood donors are often family or replacement donors, who are recruited from the relatives or friends of patients [10]. Replacement donors are described as such because for each unit of blood that is transfused, the onus is on the wider family circle to replace it in order to maintain supply [10],[11]. Paid donors may be called upon when family or friends are unable to meet this responsibility. Collecting blood from paid donors is banned in most countries in sub-Saharan Africa because they are notoriously unsafe but, in some settings without access to computerised donor registries, distinguishing them from family donors is often challenging [12].

Transfusion services in sub-Saharan Africa have been organised in this way because of culture, climate, communications, and, most importantly, because resources are limited. This fragmentation makes coordination of blood services and implementation of good laboratory practices and their quality assurance difficult, and imposes an additional burden on families in already stressful circumstances. Patients often present to hospital late in the course of their disease process [6]–[8]. Because life is at risk, the family traditionally congregates to support the patient and supplies blood when it is asked for.

If transfusion costs are not met by governments or health insurance funding, the cost of a unit of blood can often be prohibitive for families [13]. If transfusion costs are covered by governments or insurance, the cost can substantially affect the limited budgets of hospitals (for blood banks) or government (for blood centres) [10]. To produce a unit of whole blood from a family donor costs two to four times less than a unit from a voluntary non-remunerated, blood donor (VNRD) [10]. Maintaining centralised blood centres and managing the quality assurance and donor recruitment processes is expensive, so it is not surprising that, when external support is discontinued, the cost of blood products far exceeds the locally available budget.

The three principles outlined in Box 1, when imposed without careful consideration of local conditions, can undermine efforts to improve the supply of safe blood. Although the conditions of external financial assistance are discussed at the government level, practitioners on the ground are rarely consulted [14]–[17].

## Centralisation

Evidence indicates that for both severe haemorrhage and malaria-related severe anaemia, mortality increases significantly if transfusion is delayed more than 1 hour [4],[18]. Rapid availability of blood is therefore critical. However, low and unpredictable blood demand in small hospital blood banks means that they cannot justify keeping a fully tested bloodstock from family-replacement donors. It is also difficult for small blood banks to establish and maintain high-quality procedures when fewer than ten units/day are collected and there is high staff turnover [14]. Conversely, reliance on a distant centralised source for blood in settings where communications are unreliable, fuel shortages are common, and roads may be impassable, will inevitably result in delays and stock-outs.

## Sole Reliance on Volunteer Non-remunerated Donors

When applied dogmatically, the VNRD-only policy can prolong or worsen the chronic blood shortages experienced in blood systems that previously relied on family-replacement donors [7]. Neither VNRD nor family-replacement donors alone have been able to provide an adequate blood supply [19],[20], and diversity in donor sources is desirable [21]. Security of supply is difficult to achieve exclusively with VNRD who, in most countries, are predominantly secondary school students unavailable during school recesses and exam periods. Thus, the blood supply for as much as 3 months of the year is restricted. In contrast, family-replacement donors are available year round. The majority of blood donors in sub-Saharan Africa are first-time donors. Studies of HIV and hepatitis B virus prevalence in first-time donors have shown a similar risk profile in VNRD and family-replacement donors [11],[22]–[26]. Regular donors, irrespective of type, are substantially safer than first-time donors [11]. The higher costs of blood donated by VNRD compared with family-replacement donors [9],[11] result from the infrastructure necessary to recruit and retain them [20].

## Blood Component Preparation

**Figure 1:**
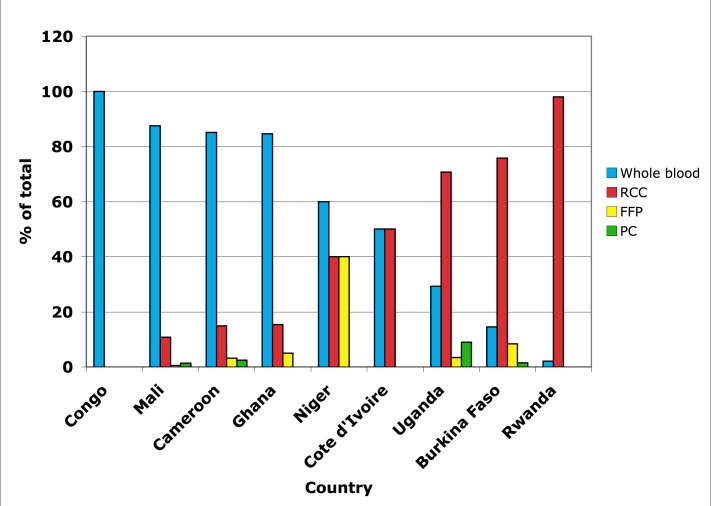
Proportion of blood and blood components prepared in nine sub-Saharan African countries. The first five countries (Congo to Niger) do not receive affluent country support, whereas the remaining four countries do. Information was assembled from references [12],[13],[27]. RCC, red cell concentrate; PC, platelet concentrate.

We recognise that the component model in the developed world has some justification from the perspectives of both quality and safety. However, in sub-Saharan Africa a policy of systematic component preparation from all donations can result in serious negative consequences (Box 2). Figure 1 illustrates the situation of individual blood centres in five countries that receive no external support for their blood services (left) and four countries that are supported by wealthy countries (right). The nationally funded blood services prepare and transfuse almost exclusively whole blood whereas the externally supported group, with the exception of one centre in Côte d'Ivoire, prepares almost exclusively red cell concentrates [27]. Only one study has presented the clinical side effects of transfusion in sub-Saharan Africa but did not compare whole blood with blood components [28].

**Box 2. Negative Outcomes of Blood Component Preparation in Sub-Saharan Africa**

- For the majority of patients in need of emergency transfusion, whole blood is more quickly available and it may be the product of choice (see above) but is actually unavailable.
- Using components instead of whole blood for emergency blood loss increases the cost of the transfusion for families or health insurance systems two- to three-fold, without clinical justification.
- The FFP removed to prepare red cell concentrates is little used clinically. Stringent regulations concerning the qualification of plasma for fractionation into intra-venous immunoglobulins, albumin, or clotting factors mean that large volumes of FFP are discarded and wasted.

## Summary and Proposals

Undoubtedly, donor countries would be concerned if the indiscriminate transposition to sub-Saharan Africa of principles underpinning transfusion services in high-income countries resulted in the undesirable outcomes described above. Clearly, such negative consequences are not the intention of these programmes, which have built and equipped large blood centres, trained a generation of transfusion specialists, and have had some success in developing VNRD programmes. However, acknowledging that these outcomes are possible and that patients' lives may be endangered warrants careful reflection and a search for alternatives. Allowing an unnecessary increase in the cost of blood relative to the rest of health care costs, in the context of an average health care budget at US$10–US$30/person/year, can undermine the fundamentals of effective blood services. In our view, reflection and systematic analysis is required to assess three key questions (Box 3).

**Box 3. Key Questions to Underpin a New Approach to Supporting Blood Transfusion Services in Sub-Saharan Africa**

- Have the imported policies had a positive or negative impact on patient outcomes and mortality? If the latter, a discourse is needed on the ethical dimensions of this issue.
- Are systems established with external aid sustainable technically and economically and do they meet the particular needs of the recipient countries?
- Should external funding for the improvement of sub-Saharan Africa blood services be linked to one set of imposed paradigms?

In the medium and long term, improving the evidence base to answer these three key questions must be a priority. There are already programmes estimating the cost-effectiveness of blood safety interventions [29], and a working party of the International Society of Blood Transfusion is dedicated to assessing different approaches to blood transfusion in developing countries [30]. On the basis of current evidence, it is suggested that leading funding organisations should focus on achieving adequate blood supply in order to save lives in the context of emergency blood transfusion in sub-Saharan Africa.

### Developing Centralised and Devolved Transfusion Services in Parallel

Centralised services bring opportunities for economies of scale and sophisticated quality assurance processes whereas hospital-based services can rapidly respond to local need and engage local communities. The absolutely critical objective of ensuring that there is enough blood available quickly wherever it is needed can be accomplished by simultaneously nurturing centralised and hospital-based transfusion services instead of investing exclusively in one or the other model [9]. For hospital-based blood banks, innovative testing and quality assurance practices adapted to low workload settings would be needed within a coordinated national blood programme. Quality assurance systems could be administered with the assistance and responsibility of the larger blood services. Technologies other than high throughput, automated, and expensive equipment with dedicated reagents would be complementary with high performance rapid tests or point-of-care technologies for example [31]. Combined systems would better serve patients' needs in most sub-Saharan Africa countries [14].

### Recruitment of Voluntary and Family-Replacement Donors

We suggest that sole reliance on blood donations from either VNRD or family-replacement donors cannot at present provide a sufficient blood supply in sub-Saharan Africa. Both types of blood donor should be welcomed as their prevalence of viral markers and background safety is similar. Both VNRD and family-replacement donors should be encouraged to become repeat donors. Paid donors should continue to be discouraged. In some countries family-replacement donors, who are neither remunerated nor coerced, are not differentiated from VNRD [32]. It is therefore proposed that all types of volunteer donors are drawn upon. While the influx of family-replacement donors can quickly reach a plateau, a volunteer donor pool is more readily expandable. Continuous recruitment efforts and innovative strategies to encourage repeat donation of all donors are urgently needed [33].

### Consideration of Whole Blood for Transfusion

The use of whole blood, particularly fresh (less than 1 week old) has been recommended not only for malaria in sub-Saharan Africa [4] but also suggested to be of equal or potentially superior clinical value for severe haemorrhage and trauma, accompanied by other medications such as tranexamic acid and appropriate surgery [34]–[36]. The preparation of components should be dictated by clinical requirements, limiting the unnecessary wastage of fresh frozen plasma (FFP). Elsewhere, the policy of separation of all donated blood into components is driven by the need for plasma for fractionation. This policy should not be imposed on countries in sub-Saharan Africa because it is inappropriate where the primary need for blood is for emergency transfusion and excess plasma cannot be used. The only plasma fractionation plant in Africa is located in South Africa and one way of reducing plasma wastage would be to set up a system that would enable it to process plasma from across Africa to produce primarily immunoglobulins but also factor VIII to treat patients with haemophilia A.

In addition to improving patient care, the changes we propose should contribute to limiting the costs of transfusion, making it more affordable and reducing the need for dependency on external funding. Efforts should be made to make tests and equipment adapted to conditions in sub-Saharan Africa [37]. While the clinical demand may well change more quickly than the economic situation, the strategy needs sufficient flexibility to accommodate developments in health care. Flexibility and pragmatism are necessary to reduce the unacceptably high rates of unnecessary deaths in Africa because blood for transfusion is lacking.

## Abbreviations

FFP: fresh frozen plasma
VNRD: voluntary non-remunerated, blood donor
